# Lysosomes, caspase-mediated apoptosis, and cytoplasmic activation of P21, but not cell senescence, participate in a redundant fashion in embryonic morphogenetic cell death

**DOI:** 10.1038/s41419-023-06326-6

**Published:** 2023-12-09

**Authors:** Cristina Duarte-Olivenza, Goretti Moran, Juan M. Hurle, Carlos I. Lorda-Diez, Juan A. Montero

**Affiliations:** Departamento de Anatomía y Biología Celular and IDIVAL, Universidad de Cantabria, 39011 Santander, Spain

**Keywords:** Apoptosis, Cartilage development

## Abstract

Micromass cultures of embryonic limb skeletal progenitors replicate the tissue remodelling processes observed during digit morphogenesis. Here, we have employed micromass cultures in an in vitro assay to study the nature of cell degeneration events associated with skeletogenesis. In the assay, “naive” progenitors obtained from the autopod aggregate to form chondrogenic nodules and those occupying the internodular spaces exhibit intense apoptosis and progressive accumulation of larger cells, showing intense SA-β-Gal histochemical labelling that strictly overlaps with the distribution of neutral red vital staining. qPCR analysis detected intense upregulation of the *p21* gene, but P21 immunolabelling showed cytoplasmic rather than the nuclear distribution expected in senescent cells. Semithin sections and transmission electron microscopy confirmed the presence of canonical apoptotic cells, degenerated cell fragments in the process of phagocytic internalization by the neighbouring cells, and large vacuolated cells containing phagosomes. The immunohistochemical distribution of active caspase 3, cathepsin D, and β-galactosidase together with the reduction in cell death by chemical inhibition of caspases (Q-VAD) and lysosomal cathepsin D (Pepstatin A) supported a redundant implication of both pathways in the dying process. Chemical inhibition of P21 (UC2288) revealed a complementary role of this factor in the dying process. In contrast, treatment with the senolytic drug Navitoclax increased cell death without changing the number of cells positive for SA-β-Gal. We propose that this model of tissue remodelling involves the cooperative activation of multiple degradation routes and, most importantly, that positivity for SA-β-Gal reflects the occurrence of phagocytosis, supporting the rejection of cell senescence as a defining component of developmental tissue remodelling.

## Introduction

Embryonic development in the animal kingdom is accounted for by massive proliferation and patterned differentiation of progenitor cells but also includes complex and regulated processes of cell death. Examples, such as the elimination of the tail during amphibian metamorphosis or the formation of free digits in tetrapods, are illustrative examples of developmental tissue remodelling processes. However, cell death participates in all embryonic processes that include cell reorganization or cell differentiation [1, 2].

The involvement of cell death in normal and abnormal embryonic development was deduced largely by histological studies using Feulgen nuclear staining and/or by staining fresh unfixed tissues with vital dyes such as Nile Blue Sulfate, Acridine Orange, or Neutral Red [3–5]. The latter is particularly useful for mapping the pattern of cell death during morphogenetic processes. Until the 1970s, the term of “necrosis” was employed to name these embryonic dying processes, and lysosomes were proposed to drive degeneration [6, 7]. However, at the end of the last century, the observation that morphologically similar cell degeneration was also a basic aspect of cancer cell biology together with the discovery of an evolutionarily conserved genetic cascade responsible for the elimination of cells destined to die changed our thoughts about the biological significance of cell death [8]. On the basis of those studies, cell death in embryonic systems began to be considered as an active and regulated process, with the term of “apoptosis” being proposed to be distinguished from “necrosis”, which was considered a passive death event [9]. A family of cysteine/aspartate proteases termed “caspases” was identified as the destructive machinery responsible for the execution of apoptosis [10]. In turn, necrosis was explained by passive rupture of cell membranes and activation of lysosomal enzymes. However, there is molecular evidence for the implication of lysosomes and active necrosis in embryonic programmed cell death [11, 12], and mouse genetic studies directed to silence components of the apoptotic molecular cascade often failed to obtain convincing phenotypes explained by the inhibition of caspase-mediated apoptosis [13–15]. In more recent studies, it was reported that embryonic remodelling processes were also associated with “cell senescence”, a process characterized by stable proliferation arrest associated with secretion of tissue-remodelling mediators and intense activation of lysosomes [16, 17].

High-density cultures (2 × 10^7^ cells/mL) of limb autopodial skeletal progenitors behave as organoids that replicate the development of the autopod-forming digits and interdigits [18]. We have previously observed that at lower cell concentrations (3 × 10^5^ cells/mL), micromass cultures of embryonic limb skeletal progenitors undergo a remodelling process that resembles the dying processes associated with digit skeletogenesis in vivo [19]. Here, we have employed the micromass assay to determine the degenerating pathways active in embryonic tissue remodelling. Immunolabelling with anti-active caspase 3, anti-cathepsin D, anti-β-Galactosidase and anti-P21 in combination with treatments with a selection of specific chemical inhibitors and with transmission electron microscopy (TEM) observations, revealed that degeneration includes a cooperative participation of caspase-dependent apoptosis, lysosomal activation, and cytosolic activity of p21 in the execution of the cell dying process. We further show that histochemical positivity for senescence-associated β-galactosidase (SA-β-Gal) marks phagosomes of cells active in the clearance of cell debris and its labelling overlaps with the traditional vital staining employed to map the areas of embryonic cell death. Remarkably, cells positive for SA-β-Gal lack specific sensitivity to the senolytic factor Navitoclax. In summary, our study confirms the cooperative activation of multiple degradation routes in the regions of programmed cell death and discards canonical cell senescence as a defining component of developmental tissue remodelling. We propose that SA-β-Gal labelling in the areas of embryonic tissue remodelling reflects the activation of an evolutionary primitive phagocytic defence process that is distinct, but shares several features with cell senescence.

## Materials and methods

Fertilized Rhode Island chicken eggs obtained from a regular commercial supplier (Granja Santa Isabel. Cordoba, Spain) were incubated at 38 °C. At the desired stage the eggs were opened, and the hind limbs were microdissected for further processing. The embryo extraction was performed following the ethical recommendations of the European Communities Council.

### Micromass culture and tissue processing

The undifferentiated tissue of the distal margin of the limb (progress zone) that contains “naive” skeletal progenitors [20] was dissected free from the leg buds of HH-stage 25 embryos (4.5 id) and cells were dissociated by digestion with 0.25% trypsin (Sigma) and 0.25% collagenase (Worthington). After filtering through a 70-μm strainer (Miltenyi Biotec), the cells were resuspended in Dulbecco’s modified Eagle medium (DMEM, Lonza) with or without 10% FBS. Ten microlitre drops were pipetted into each well of a 48‐well plate (Thermo Scientific). Most experiments were performed using a concentration of 3 × 10^5^ cells/mL, but we employed cell concentrations up to 1.1 × 10^7^. Cells were allowed to attach for 2 h, and then 200 μL of DMEM was added to each well.

### Morphological and immunohistochemical studies

SA-β-Gal was analysed histochemically at pH 6 in cultures fixed in 4% glutaraldehyde as described by Debacq-Chainiaux et al. [21]. Whole limb buds at different developmental stages were fixed in the same fashion, sectioned with a vibratome, and processed for comparative microscope studies (*n* = 12). The number of SA-β-Gal-positive cells in controls and treated micromasses was measured in five cultures, performed in independent days, by counting with ImageJ. For this purpose, we selected a surface of 1 mm^2^ in the centre of the micromass culture. “Blind researcher” not involved with the experiment made the measurements. Considering the characteristics of the sample set, data were analysed using the *F*-test followed by Student’s *t* test. Statistical significance was set at *p* < 0.05.

Immunolabelling was performed in cultures grown in fibronectin-coated coverslips and fixed in 4% paraformaldehyde (PFA). Small samples of squashed interdigital tissue were also employed for comparative purposes of in vivo and in vitro conditions. The following antibodies were employed: anti-β-galactosidase monoclonal antibody (ab116, kindly provided by Dave Sambhav from Abcam Inc.); anti-cathepsin D polyclonal antibody (sc-6486; Santa Cruz Biotechnology); anti-p21^WAF1/CIP1^ polyclonal antibody (PA5-34805; Thermo Fisher); anti-p21 monoclonal antibody (sc-817; Santa Cruz Technology); anti-active caspase 3 polyclonal antibody (AF835; R&D Systems and Biothechnology); and the chick macrophage marker TAP1 (Developmental Studies Hybridoma Bank). Polyclonal anti-SOX9 antibody (AB5535; Merck-Millipore) and rhodamine-phalloidin (P1951; Sigma) were employed for nuclear and cytoplasmic counterstaining, respectively, when needed. Observations were made with an LSM510 laser confocal microscope (Zeiss) employing a minimum of 10 independent samples for each antibody or combination of antibodies.

Apoptotic cell death was analysed by TUNEL assay, using the in situ cell death detection kit (Roche). The estimation of the number of double-positive cells for TUNEL and P21 or cathepsin D was carried out by counting the double positive cells out of a total of 100 TUNEL-positive cells from four micromasses performed in independent days, that were double labelled (TUNEL/P21 and TUNEL/cathepsin D).

Neutral red vital staining was performed following the method of Hinchliffe and Ede [22]. The culture medium of the micromasses was substituted by a solution of 1 × 10^−5^% of Neutral Red in PBS and maintained in the incubator at 38 °C for a few minutes (10–15 min). Staining was controlled under the binocular microscope and photographed. In a number of cases (*n* = 10), once photographed, the cultures were washed in PBS and fixed for subsequent detection of SA-β-Gal.

### Transmission electron microscopy (TEM) and histology

For TEM and histology, micromasses were fixed for 4 h at room temperature in 4% glutaraldehyde in phosphate buffer. After fixing, the cultures were carefully detached from the wells, postfixed in 2% osmium tetroxide, dehydrated in ethanol and embedded in araldite. Sections (1 μm thick) were obtained and stained with toluidine blue. Ultrathin sections were stained with lead citrate and examined with a Philips EM208 electron microscope. For this purpose, we used 3 samples grown for 2 and 3 days, respectively, using cultures obtained from 3 independent experiments.

### Treatments

The involvement of caspase-dependent apoptosis, cathepsin D, P21, and cell senescence was analysed using a pharmacological strategy. Caspase activation was prevented with Q-VD-OPh (20 µM), an efficient and selective broad-spectrum caspase inhibitor [23]. Lysosomal cathepsin D was inhibited with pepstatin A (Sigma-Aldrich, Deisenhofen, Germany). UC2288 (Merk) was employed as a p21 inhibitor that attenuates p21 expression at both at the transcriptional and protein levels and lacks effects on AKT and ERK signalling pathways [24]. The implication of senescent cells in tissue remodelling was analysed by administration of the senolytic agent Navitoclax, that promotes the elimination of senescent cells [25].

All the employed drugs were diluted in DMSO that was added at the same concentration as the medium of control cultures.

In all cases, treatments were performed overnight (12 h) at the end of the second day of culture (36 h). Changes in the intensity of cell death were next evaluated by flow cytometry in a minimum of 6 independent experimental samples.

### Flow cytometry

Control and treated cultures were dissociated with trypsin EDTA (Lonza) and fixed in 90% ethanol. One million cells were used in each test. Samples were incubated overnight at 4 °C with 0.1% sodium citrate, 0.01% Triton X-100, and 0.1 mg/mL propidium iodide (Sigma) and subjected to flow cytometry analysis in a Becton Dickinson FACSCanto cytometer. Considering the characteristic of the sample set, data were analysed using ANOVA followed by Bonferroni test for post hoc comparisons. Statistical significance was set at *p* < 0.05.

### Real-time quantitative PCR (qPCR) for gene expression analysis

Gene expression analysis was performed by SYBR Green qPCR as described previously [19], employing *Gapdh* as a normalizer. The following genes were explored (primers are shown in Supplementary Table 1): cell cycle regulators previously associated with senescence and apoptosis, including *p21*, *p16*, *p53*, *p63* and *p73*; lysosomal components, including *cathepsin D* and *galactosidase beta 1* (*GLB1*) genes; autophagic genes, including *ATG5* and *ATG7*; immunocytokines, (*IL6*); cell death genes, including antiapoptotic *Bcl2*, and proapoptotic *Bak1*; and a panel of SASP members (https://senequest.net/), including: members of the insulin-like growth factor gene family (*Igf1*, *Igfbp5*, and *Igfbp7*), hepatocyte growth factor (*HGF*), and the extracellular matrix proteases *MMP2* and *MMP9*. Mean fold change values were calculated for each gene using at least five independent samples obtained from 4 micromass cultures each. The relative expression level was evaluated according to the 2^−(∆∆Ct)^ equation [26]. Considering the characteristic of the sample set, data were analysed using the *F*-test followed by Student’s *t* test, and the statistical significance was set at *p* < 0.05.

## Results

**Fig. 1:**
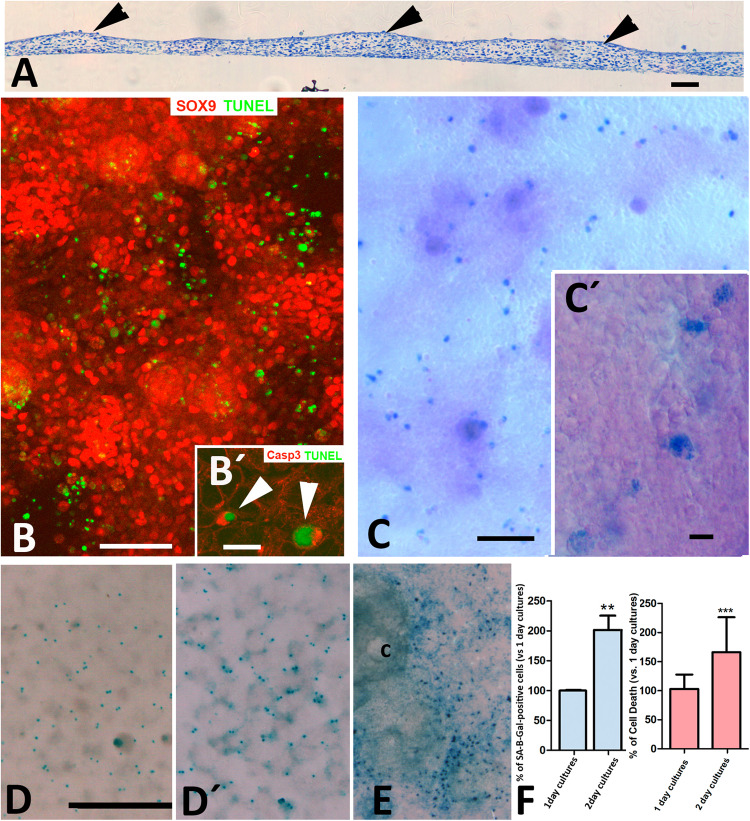
Apoptotic and SA-β-gal-positive cells in micromass cultures. **A** Semithin section of a micromass cultured for 48 h at low magnification to show the initial appearance of thickened regions (arrow heads) that precede the formation of cartilage nodules. **B** Confocal microscope image of a 3-day-old micromass double labelled for SOX9 (red) and TUNEL(green) showing the preferential arrangement of TUNEL-positive apoptotic cells around cell aggregates that are highly positive for SOX9. Insert B´ shows double labelling for TUNEL (green) and active caspase 3 (red) to illustrate the intense positivity of apoptotic cells for active caspase 3 (arrows). **C** SA-β-gal histochemical staining counterstained with eosin, showing the preferential distribution of SA-β-gal-positive cells in the internodular regions in a 3-day-old micromass. Insert **C´** is a detailed view of SA-β-gal-positive cells illustrating their increased size and foamy appearance. **D**, **D´** Low magnification of control micromasses after 24 h (**D**) and 48 h (**D´**) of culture after SA-β-gal histochemistry. **E** Domain of SA-β-gal-positive cells around differentiating cartilage nodules (c) in a micromass elaborated with 11×10^5^ cells/µl, cultured for 7 days. **F** Graphic representations of the relative number of SA-β-gal-positive cells (blue) and dead cells (red) in 2-day cultures vs Day 1. The number of cells on Day 1 was considered 100%. Magnification bar in **A** = 30 µm; bar in **B** = 100 µm; bar in **B´** = 10 µm; bar in **C** = 300 µm; bar in **C´** = 25 µm; bar in **D**, **D´**, **E** = 1 mm. Statistical significance: ***p < 0.001; **p < 0.01.

Cultures of embryonic limb autopod skeletal progenitors at high density recapitulate the structural events associated with the formation of digits in an organoid-like fashion [18, 19]. At very high cell concentrations the cultures develop digits and interdigit regions [18]. At concentration of 3 × 10^5^ cells/ml (termed micromass cultures), progenitors develop in an irregular fashion forming thickened nodular regions that progressively deposit cartilage extracellular matrix surrounded by thinner internodular regions where cells remain undifferentiated (Fig. 1A). The nodular regions were highly positive for SOX9 and differentiated into cartilage, while internodular regions showed lower positivity for this cartilage marker (Fig. 1B). TUNEL-positive apoptotic cells displayed a preferential internodular distribution (Fig. 1B). As expected, TUNEL positive cells exhibited intense labelling for active caspase 3, which is a characteristic marker of apoptotic cell death (Fig. 1B´). Histochemical detection of SA-β-Gal (pH 6) identified a subpopulation of large cells of foamy appearance (Fig. 1C) that contrasted with a majority population of smaller round eosinophilic cells. Due to the elevated expression of *p21*, and the large size (Fig. 1C´), the SA-β-Gal positive cells were previously described as senescent cells [19]. The number of these cells increased in parallel with the increase in the number of apoptotic cells, becoming duplicated on the second day of culture (Fig. 1D, D´, F). Furthermore, when using very high cell concentration cultures (1.1 × 10^7^; *n* = 6), elongated cartilages were formed [18], between which SA-β-Gal cells formed well-defined domains reminiscent of interdigits (Fig. 1E).

**Fig. 2:**
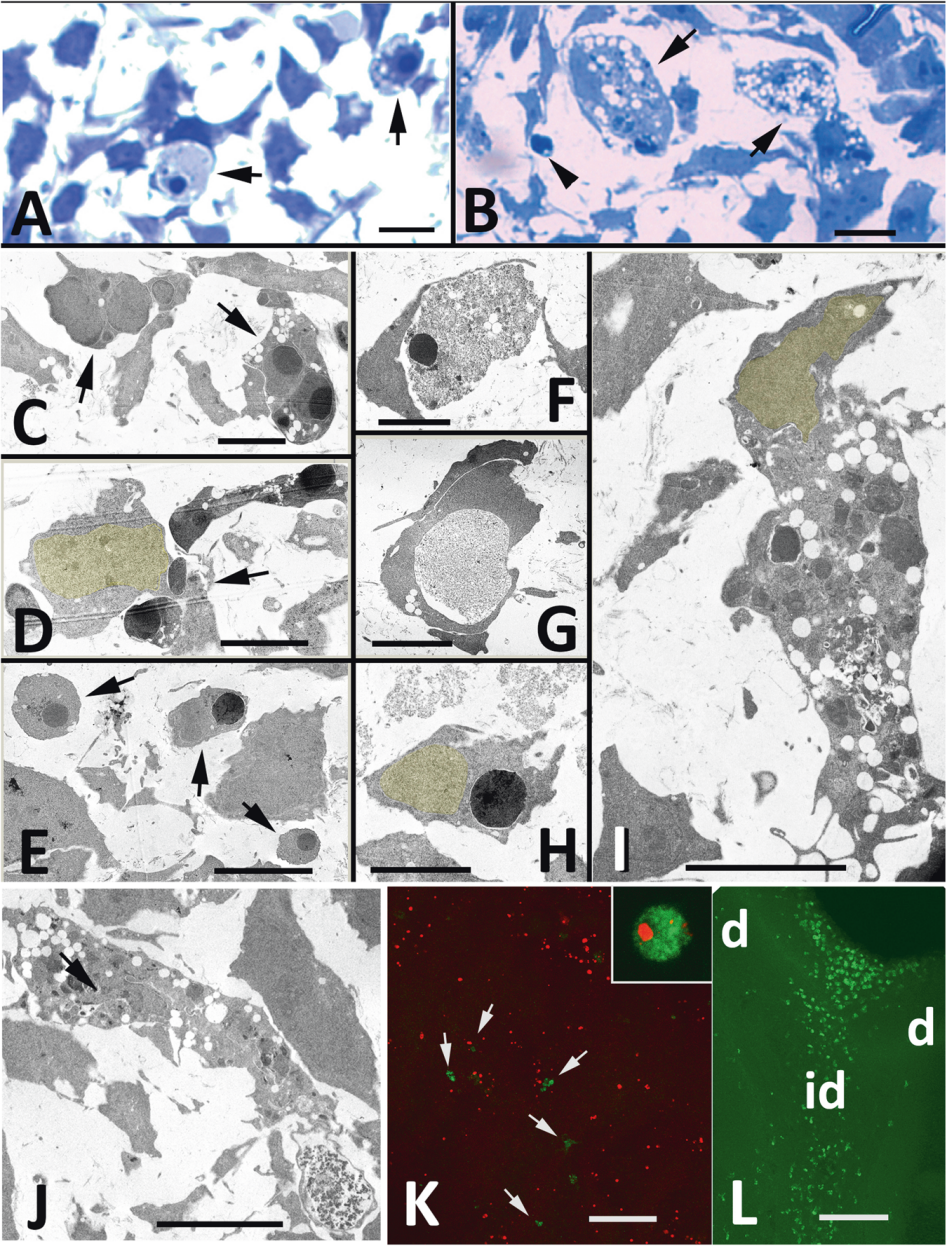
Morphological and structural characterization of degenerating cells. **A**, **B** Semithin sections of 72 h micromass cultures stained with toluidine blue. **A** Isolated dead cells (arrows). **B** shows two large vacuolated cells (arrows) and an isolated apoptotic fragment (arrowhead). **C** TEM micrograph showing two characteristic apoptotic cells (arrows) free in the extracellular space. **D** shows an apoptotic cell in course of fragmentation. Note the close association with the healthy neighbouring cells (arrow). **E** Apoptotic cell fragments (arrows) free in the extracellular space. **F**, **G** Degenerated cell fragments during the course of internalization by neighbouring cells. Note the necrotic appearance of the dead cell in (**F**). **H** Mesodermal progenitor showing a phagosome containing nuclear material. Note that the nucleus of the phagocytic cell is enhanced in yellow. **I** Large vacuolated cell containing multiple phagosomes. The nucleus is enhanced in yellow. **J** Large vacuolated cell containing multiple phagosomes. Note the chromatin condensation of the nucleus (arrow) compared with the nucleus in (**I**). **K** Low magnification view showing numerous apoptotic cells TUNEL-positive (red labelling) and very scarce macrophages positive for TAP1 (green labelling) in a 48 h micromass culture. The inset is a detailed view of a large cell TAP1 positive (green) showing TUNEL positivity (red). **L** TAP1-positive macrophages (green labelling) in the regressing interdigits (id) of a 7.5-day-old embryo. Compare the density of macrophages in vivo with that observed in the remodelling micromass culture (**K**). Digits 3 and 4 are indicated by d. Magnification bar in (**A**, **B**) = 10 µm; bar in (**C**–**J**) = 5 µm; bar in (**K**) = 100 µm; bar in (**L**) = 150 µm.

Semithin sections stained with tolouidine blue confirmed the presence of isolated dead cells (Fig. 2A) and large vacuolated cells containing phagosomes filled with cell remnants (Fig. 2B) in the internodular regions. The observations under TEM enabled the identification of three distinct “degenerating” events, including (i) canonical apoptotic cells in course of fragmentation (Fig. 2C, D, E); (ii) apoptotic cell fragments undergoing secondary necrotic degeneration in course of phagocytosis (Fig. 2F, G); and (iii) phagocytic cells containing few (Fig. 2H) or multiple phagosomes (Fig. 2I, J) filled with remnants of dead cells. Among the large phagocytic cells, the presence of intense vacuolar degeneration and chromatin condensation indicative of cell death was remarkable (Fig. 2J). The distribution and appearance of these phagocytic cells corresponded with the large vacuolated cells present in the semithin sections (Fig. 2B) and with the large SA-β-Gal-positive cells observed in the histochemical study.

To ascertain whether phagocytic cells are specialized macrophages, we combined a TUNEL assay with immunolabelling for the chick macrophage marker TAP1 [27]. As shown in picture (Fig. 2K, L), in contrast with the abundance of macrophages in the interdigits in vivo (Fig. 2L), the number of cells positive for TAP1 in the internodular regions was scarce in relation to the number of apoptotic cells (Fig. 2K). Consistent with TEM observations, some of TAP1-positive macrophages were apoptotic (Fig. 2K).

**Fig. 3:**
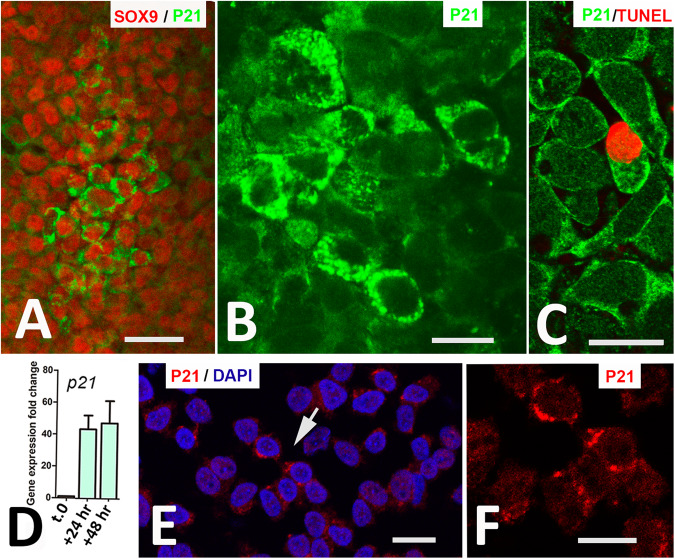
Analysis of P21 expression. **A** Double immunolabelling for SOX9 (red) and p21^WAF1/CIP^ (green) showing a clump of progenitors highly positive for P21 in a 2-day-old micromass culture. **B** Detailed view of progenitors showing intense cytoplasmic positivity for P21^WAF1/CIP^. **C** Double labelling for TUNEL (red) and P21^WAF1/CIP^ (green) showing cytoplasmic P21 in a TUNEL-positive (red) apoptotic cell. **D** Graphic representation of p21 gene expression fold changes in progenitors at time 0 and after 24 and 48 h of culture. **E** Interdigital tissue squash on Day 7 of incubation showing cytosolic P2-positive immunolabelling (red) and DAPI nuclear staining (blue). **F** is a detailed view of the red channel of cells indicated by arrows in (**E**), showing the cytoplasmic distribution of P21 labelling. Magnification bar in (**A**) = 20 µm; bar in (**B**, **C**, **E**, **F**) = 10 µm.

To further investigate whether the large SA-β-Gal positive cells are senescent, we performed an immunocytochemical and transcriptional analysis of the expression pattern of p21 as a central regulator of cell senescence [28]. Immunolabelling for P21, detected clusters of cells with intense cytoplasmic positivity dividing into nodules the primitive aggregates of cells positive for SOX9. Remarkably, these cells lacked the nuclear distribution of P21 associated with the regulation of cell cycle in senescent cells (Fig. 3A, B). As shown in Fig. 3B, C, cells with high cytoplasmic P21 immunolabelling, included healthy cells and also 45% of the cells TUNEL-positive (*n* = 100). The increased expression of P21 in the micromass was detected by qPCR from time 0 to day 2 of culture (Fig. 3D). Importantly, P21 also showed a preferential cytoplasmic localization in the developing limb interdigits in vivo, although labelling intensity was lower than in the cultured progenitors (Fig. 3E, F), indicating that this is a physiological feature in the areas of programmed cell death.

### Transcriptional regulations

As shown in Table 1, from Day 0 to Day 1 of culture a significant number of genes, belonging to the senescence-associated secretome phenotype (SASP) [29], appeared upregulated including the lysosomal components *GLB1*, *cathepsin D*, and *MMP2*, and the secreted growth factor *IGF1* and the insulin-like growth factor binding proteins *IGFBP5* and *IGFBP7*. *p21*, which is a key element of developmental senescence, was also intensely upregulated (40×). *p16*, that is also a negative cell cycle regulator involved in cell senescence appeared upregulated but its expression level was extremely low, as deduced by Ct values over 34 (in contrast with *GAPDH* Ct value = 18; and *p21* Ct value = 25). The same happened for the cytokine *IL6*, which is a characteristic member of the *SASP*. Transcription factors of the *p53* family of tumour suppressor genes, that negatively regulate the progression of the cell cycle, including *p63*, and *p73* [30], were downregulated, while p53 did not appear regulated at statistically significant levels (Table 1).

**Table 1:** Transcriptional modifications after 24 h of culture of limb skeletal progenitors.

Transcriptional regulations			
Gene	+24 h	Gene	+24 h
Lysosomal components		Death genes	
Ch. Cathepsin D	4.30 ± 1.12***	Ch. Bak1	2.77 ± 0.34**
Ch. GLB1	3.81 ± 1.29**	Ch. Bcl2	1.90 ± 0.70*
Ch. MMP2	11.84 ± 4.85*	Cell cycle regulators	
		Ch. p21	40.42 ± 20.69***
Ch. MMP9	3.44 ± 3.49		
		Ch. p16^a^	3.19 ± 0.95*
Growth factors and cytokines			
Ch. IGF1	31.70 ± 11.43***	Ch. p53	0.78 ± 0.36
Ch. IGFBP5	29.31 ± 10.34**	Ch. p63	0.33 ± 0.09***
Ch. IGFBP7	9.06 ± 2.87**	Ch. p73	0.41 ± 0.13***
Ch. HGF^a^	0.71 ± 0.29	Autophagic inducers	
		Ch. ATG5	0.57 ± 0.28*
Ch. IL6^a^	25.27 ± 11.05*		
		Ch. ATG7	1.76 ± 0.29*

The values are compared with expression at *t* = 0 h.

***p < 0.001; **p < 0.01; *p < 0.05.

^a^Extremely low expression.

In the apoptotic molecular cascade, the pro-apoptotic BH-multidomain gene *Bak1* appeared upregulated (2.7×) while the anti-apoptotic *Bcl2* gene was upregulated at lower levels (1.9×). The autophagic-inducer gene *Atg5*, appeared to be downregulated in contrast *Atg7* was slightly upregulated.

Other studied genes associated with cell senescence not regulated in this initial period of culture are included in Table 1.

### Positive immunolabelling for lysosomal enzymes

**Fig. 4:**
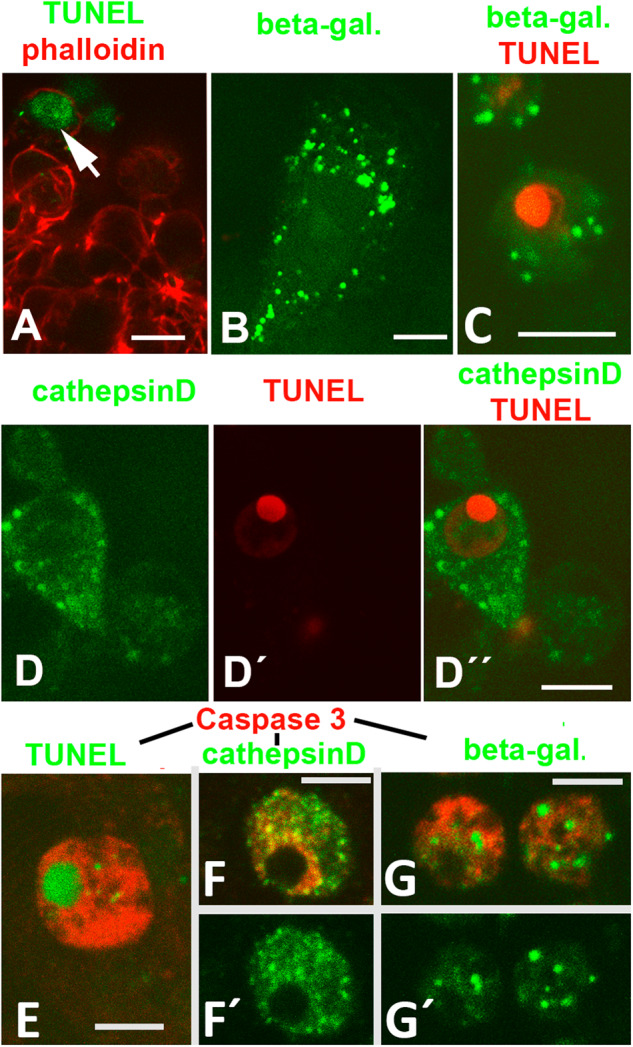
Confocal images showing the association of TUNEL and lysosomal positivity in 2 and 3-day micromass cultures. **A** TUNEL (green labelling) positive cells in the periphery of a nodular prechondrogenic aggregate. The sample was counterstained with phalloidin (red). **B** Detailed image of an internodular cell highly positive for beta-galactosidase (green labelling). **C** Internodular apoptotic body showing TUNEL (red labelling) and beta-galactosidase (green labelling) positivity. **D**–**D´´** TUNEL-positive cells (red labelling) with intense cytoplasmic cathepsin D labelling (green labelling). **D** Green channel (cathepsin D); **D´** red channel (TUNEL); and **D´´** merged image. **E** Double labelling for TUNEL (green) and active caspase 3 (red) illustrating positivity of apoptotic cell fragments for active caspase 3. **F**, **F´** Double labelling for active-caspase 3 (red) and cathepsin D (green). **F** Merged image; **F´** green channel. **G**, **G´** Double labelling for active caspase 3 (red) and beta-galactosidase (green). **G** Merged image; **G´** green-channel. Magnification bars in (**A**, **B**, **D**) = 8 µm; bar in (**C**, **E**, **F**) = 5 µm; bar in (**G**) = 10 µM.

Further characterization of the remodelling process was performed by confocal microscopy of cultures double labelled for apoptosis (TUNEL) and canonical lysosomal markers (β-galactosidase; and cathepsin D). TUNEL analysis confirmed the abundance of TUNEL/active-caspase 3 positive apoptotic cells in the internodular spaces of micromass cultures (Fig. 4A). In the same fashion, cells positive for β-galactosidase (Fig. 4B, C) and cathepsin D (Fig. 4D–D´´) immunolabelling were identified in the internodular regions of the micromass. Double labelling with TUNEL and the lysosomal markers, revealed intense lysosomal positivity in most TUNEL cells (78 out of 100 TUNEL-positive cells; Fig. 4C, D).

In agreement with these findings, TUNEL-positive cells (Fig. 4E) showed double positivity for active-caspase 3 and cathepsin D or β-galactosidase (Fig. 4F, G). The size of the lysosomal-positive clumps in these cells supported their association with active phagocytic processes.

### Overlapping distribution of Neutral red vital staining and SA-β-Gal histochemistry

**Fig. 5:**
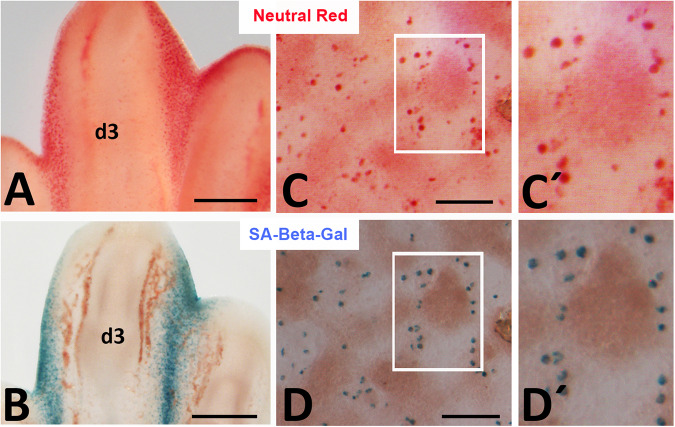
Overlapping pattern of Neutral red vital staining and SA-β-Gal histochemistry. **A**, **B** Detailed images of digit 3 (d3) and the adjacent second and third interdigital spaces of the limb bud on Day 8 of incubation (32HH) illustrating the similitude between neutral red vital staining (**A**) and SA-β-Gal histochemistry (**B**). **C**, **D** Detailed micrographs of a 3-day-old micromass culture vitally stained first with neutral red (**C**) and second, following SA-β-Gal histochemistry after being fixed with glutaraldehyde at pH 6 (**D**). **C´**, **D´** are amplified details of the region framed in white in (**C**, **D**). Note the overlapping labelling pattern obtained with both procedures. Magnification bars = 250 µm (**A**, **B**); 150 µm (**C**, **D**).

Considering that cultures replicate the degenerative events occurring during the formation of the digits in vivo (Fig. 5A, B), we wanted to compare the pattern of neutral red vital staining, as a canonical method to map areas of cell death [3], with the pattern of SA-β-Gal histochemistry, via a double labelling approach. As expected, vital staining with neutral red revealed positive cells distributed throughout the internodular regions (Fig. 5C, C´). To overcome the difficulty of double labelling procedures (vital staining requires unfixed live tissue) we performed a sequential labelling protocol. We processed the cultures first for vital dye staining (Fig. 5C, C´) followed by a quick fixation with glutaraldehyde at pH 6 and subsequent histochemical detection of SA-β-Gal (Fig. 5D, D´) and the staining patterns of the same microscopic fields were compared. As shown in Fig. 5C, D, neutral red vital staining (Fig. 5C, C´) overlapped with SA-β-Gal positive cells (Fig. 5D, D´). The pattern of SA-β-Gal histochemical labelling was not modified when staining with neutral red was omitted.

### Decreased cell death following chemical inhibition of caspases, lysosomal enzymes, and p21

The functional significance of lysosomes, caspases, and P21 in the dying process was next analysed by pharmacological inhibition experiments. In all cases, treatments were performed for 12 h at the end of the second day of culture and cell death was evaluated by propidium iodide flow cytometry. The number of SA-β-Gal positive cells was evaluated in histochemically processed samples with the help of the imageJ.

**Fig. 6:**
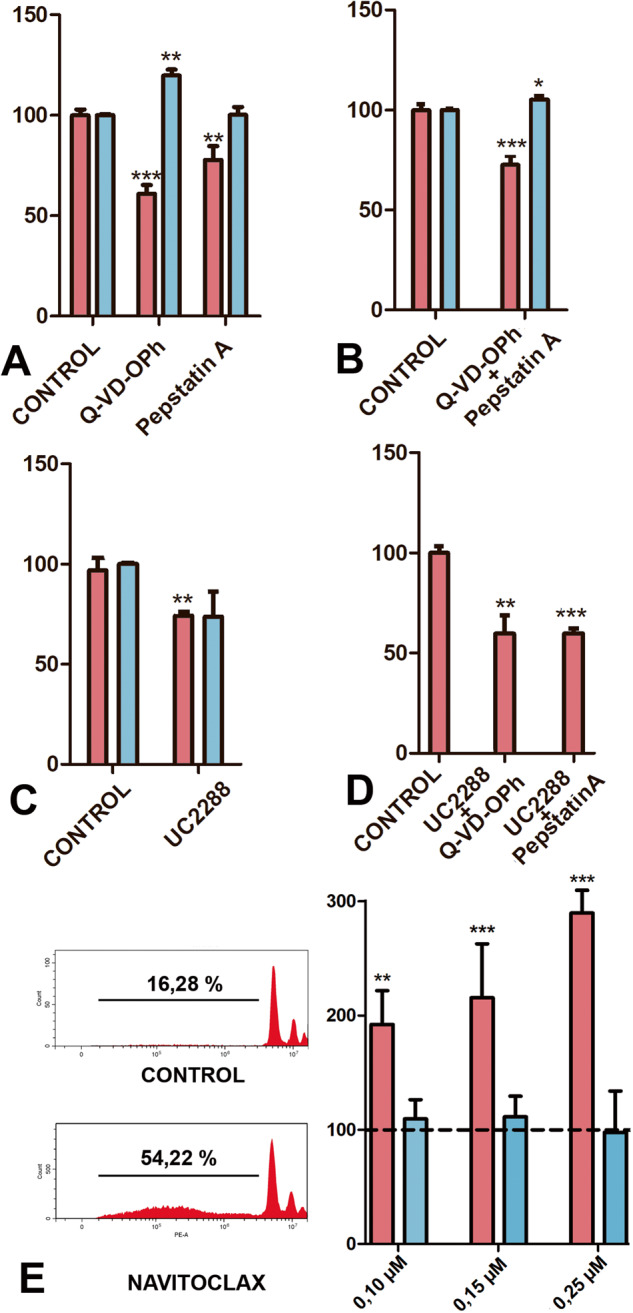
Effects of different inhibitors on micromass cultures. In all graphs, the percentage of dead cells is represented by red columns, and the percentage of SA-β-Gal-positive cells is represented by blue columns. Treatments were administered for 12 h after 30 h of culture. The values in control micromasses (treated only with drug vehicle) were considered 100%. Cell death was measured by flow cytometry using propidium iodide. SA-β-Gal-positive cells were quantified by histological examination. **A** Bars from left to right respectively represent micromasses treated with control conditions; 20 µM Q-VD-OPh; 5 µM Pepstatin A; **B** combination of 20 µM Q-VD-OPh plus 5 µM Pepstatin A. **C** Cultures treated with 5 µM UC2288. **D** Cultures treated with 5 µM UC2288 plus 20 µM Q-VD-OPh (left bar), and 5 µM UC2288 plus 5 µM Pepstatin A (right bar). **E** Representative flow cytometry plots (propidium iodide) of control and 0.25 µM Navitoclax treated cultures showing differences in the intensity of death cells (left images). Bars show the fraction of apoptotic cells with fragmented DNA in the treated and control cultures. The image on the right is a graphic representation of the incidence of dead cells (red columns) and the number of SA-β-Gal-positive cells in cultures treated with 0.10 µM, 0.15 µM, and 0.25 µM Navitoclax. The dotted line represents values in control conditions. Statistical significance: ***p < 0.001; **p < 0.01; *p < 0.05.

Q-VD-OPh (20 µM), which is a selective broad-spectrum caspase inhibitor [23], caused 40% inhibition of cell death versus untreated controls (Fig. 6A). This inhibition was accompanied by a moderate but significant increase in the number of SA-β-Gal-positive cells (Fig. 6A), suggesting that these cells die via caspase activation. Pepstatin A (5 μM) caused a 27% reduction in cell death (Fig. 6A), without changing the number of SA-β-Gal-positive cells. The combination of both inhibitors had not additional effect on cell death and SA-β-Gal-positive cells compared to single treatments (Fig. 6B).

Inhibition of p21 was performed with 5 µM UC2288, which selectively decreases mRNA and cytosolic protein levels [24]. Analysis at the end of the treatment resulted in a moderate but significant reduction in cell death (Fig. 6B) without significant changes in the number of SA-β-Gal-positive cells. When UC2288 treatments were combined with Pepstatin A or Q-VD-OPh, cell death was unchanged in comparison to single treatments (Fig. 6D).

### Navitoclax treatment increased cell death without changing the number of SA-β-Gal cells

Navitoclax is a senolytic agent that promotes the elimination of senescent cells in cancer and degenerative diseases by targeting prosurvival members of the *Bcl2* gene family [31] and is active in senescent embryonic fibroblasts [25]. Here, treatments with Navitoclax were performed for 12 h after 36 h of culture, employing concentrations of 0.10, 0.15, and 0.25 µM. The number of dying cells evaluated by flow cytometry increased in a dose-dependent fashion by the addition of Navitoclax to the culture medium but the number of cells SA-β-Gal-positive was not significantly modified (Fig. 6E). These findings discard the specificity of SA-β-Gal-positive cells to the pro-apoptotic influence of Navitoclax, which is a characteristic feature of senescent cells.

## Discussion

Embryonic development is accompanied by regulated tissue remodelling processes that account for the morphological shaping and structural differentiation of embryonic organs [1, 32]. Here, we employed micromass cultures of embryonic autopodial limb skeletal progenitors as an organoid-like in vitro assay that replicates the cell death-mediated remodelling process that accompanies skeletogenesis and individualization of the digit in vivo [18, 19]. Our study addressed two major questions concerning “embryonic programmed cell death”: (i) first, the mechanistic basis of the dying process; and (ii) second, the proposed involvement of cell senescence in embryonic tissue remodelling associated with morphogenesis [16, 33].

The canonical view of embryonic cell death in vertebrates is that dying cells are predominantly executed via apoptosis in a caspase-dependent fashion. In our assay, and also in the embryo, dying cells displayed the characteristic morphology of apoptotic cells [7]. However, cathepsin D has been identified as an apoptotic triggering factor [34], and we have previously observed that during digit morphogenesis, interdigital cell death involves not only the activation of caspases but also the delivery of lysosomal enzymes accompanied by a decrease in the pH value of the regressing tissue [35–37]. In addition, genetic studies in mice failed to block interdigital cell death by silencing initiator or executioner caspases [13–15] or lysosomal cathepsin D [38]. Our present observations revealed an intense upregulation of lysosomal enzymes and active caspase 3, and showed that chemical inhibition of either caspases or cathepsin D very significantly decreased the intensity of cell death, but treatments combining both inhibitors failed to potentiate the inhibition of each factor administered separately. These findings are consistent with the genetic studies mentioned above, showing that caspases and lysosomal enzymes are redundant mediators of cell death. These findings together with the results obtained in genetic studies, suggest that the confluence of multiple degenerative routes is a defining aspect of programmed cell death in developing vertebrates [2, 6].

Our study reveals the involvement of *p21* in programmed cell death. The cytosolic distribution of P21 in the cultured progenitors and in the interdigital mesoderm in vivo supports this interpretation. P21 is a multifunctional factor with important roles, not only in the control of the cell cycle and cell differentiation but also in the positive and negative control of apoptotic cell death and autophagy [39–41]. The control of the cell cycle occurs at the nuclear level by inhibition of cyclin-dependent kinases and PCNA-dependent DNA polymerase blockade [40]. In contrast, the function of P21 in the control of cell death has been associated with its cytoplasmic localization [41, 42]. The mechanisms by which P21 promotes cell death remain to be clarified [40], but in human hepatocarcinoma, it was found that P21 promotes apoptosis by modulating the Bcl2:Bax ratio [43]. Here, we have observed that the upregulation of *p21* in the cultures correlates with the upregulation of *Bak1*, which is the BH-multidomain proapoptotic member of the BCL gene family in chickens and is responsible for the permeabilization of the outer mitochondrial membrane in the apoptotic cascade. The absence of potentiation of the anti-cell death effect of pancaspase inhibitor (Q-VD-OPh) or a cathepsin D inhibitor (Pepstatin A) in combination with *p21* silencing, is again consistent with the downstream convergence of degenerative routes in the execution of cell death, which appears to be triggered by initial DNA damage [44].

In recent decades, it has been reported that tissue remodelling involves, not only cell death but also cell senescence that was termed “developmental senescence” [16, 29, 33]. Our present findings in the micromass assay argue against the involvement of “canonical” cell senescence in embryonic cell death remodelling processes. Cell senescence is a defence mechanism of major importance in cancer, ageing, and degenerative diseases [45, 46]. However, cell senescence includes distinct stages and types [47] with variations in molecular signatures and distinct specialized functions [48, 49]. In fact, there is not a single specific marker to unequivocally identify cell senescence in the tissues [17, 49, 50], and its diagnosis relies on the association of different biomarkers, including (i) stable cell cycle arrest; (ii) positivity for beta-galactosidase at pH 6, termed SA-β-Gal; (iii) production of characteristic secretome, collectively termed the senescence-associated secretory phenotype (SASP); (iv) transcriptional upregulation of tumour suppressor genes, with *p16* and *p21* being the most representative; (v) multiple morphological features, such as increased cell size, cytoplasmic granularity or increased heterochromatin; and (vi) apoptotic susceptibility to senolytic drugs.

SA-β-Gal reflects an increase in lysosomal mass characteristic of senescent cells [51] but most likely also marks other cells [52] rich in lysosomal enzymes, such as macrophages. The presence of domains of SA-β-Gal-positive cells was considered in previous studies to reflect a cell senescence process associated with tissue remodelling [16, 19, 33, 53]. This interpretation was supported by accompanying features, including the upregulation of a SASP secretome and *p21*. Our present observations question the interpretation of cell senescence based on SA-β-Gal-positive labelling only. First, our TEM study shows that the structural components of the micromasses include large vacuolated cells, some of which are identifiable as mature TAP1-positive macrophages. The morphology of these large phagocytic cells together with the intense immunolabelling for β-galactosidase enables their identification as the SA-β-Gal-positive cells observed in the histochemical assay. Furthermore, the overlapping distribution of SA-β-Gal histochemical positivity with the pattern of neutral red vital staining, confirmed that this senescent biomarker [21, 54], marks the phagosomes associated with the clearance of dead cells [55]. For decades vital staining with Neutral Red or Nile Blue Sulfate, which are lysosomotropic dyes [56], has been employed as a marker of embryonic cell death, assuming the implication of lysosomes in the dying process [4]. Taking into account the abovementioned discussion, it is likely that the increase in cells positive for SA-β-Gal in the course of the cultures could be explained by the maturation of premacrophages present in the embryonic tissue [57–59]. The upregulation of *p21* in the areas of tissue remodelling together with a mild limb phenotype of mice deficient for *p21* was claimed to support the implication of developmental senescence in the embryonic remodelling processes [16, 33]. However, as mentioned above, P21, instead of displaying the nuclear distribution required to block proliferation, it shows a precise cytoplasmic distribution both in the cultures and in the remodelling interdigits in vivo. Furthermore, the implication of p21 in the control of cell death discussed above might also contribute to explaining the mild phenotype of mice subjected to *p21* silencing.

A final piece of evidence supporting the idea of rejecting the significance of cell senescence in embryonic tissue remodelling was the results of treatments with the senolytic drug Navitoclax [25]. Senolytic drugs are proapoptotic factors that kill nonproliferating senescent cells resistant to other cytotoxic factors employed in chemotherapy. However, in our study, Navitoclax treatments resulted in an unspecific killing effect on the cultured progenitors while the number of SA-β-Gal-positive cells was not significantly modified.

Taking together all the above discussed observations, our findings support the idea that SA-β-Gal labelling in the developing system reflects the activation of phagocytosis, as a part of the innate immunity, required for the clearance of cell remnants derived from the degenerative process, thus cooperating with apoptosis in sculpting embryonic structures [53]. The SASP components upregulated in the culture included inflammatory cytokines, matrix metalloproteases, and growth factors. Remarkably, all these components may be produced by the phagocytic cells [60–62], which is consistent with an active role of phagocytic cells not only in the clearance of apoptotic fragments but also in remodelling the extracellular matrix. The phagocytic nature of SA-β-Gal positive cells was proposed in a preprint manuscript by Hernandez-Garcia et al. [63]. In summary, our observations suggest that SA-β-Gal labelling in the areas of embryonic tissue remodelling reflects the activation of an evolutionary primitive defence process that shares several features with cell senescence.

### Supplementary information

Supplementary Table 1

Reproducibility checklist

## Data Availability

The datasets generated and/or analysed during the current study are available from the corresponding author on reasonable request.
