# Supplementary material for: Lysosomes, caspase-mediated apoptosis, and cytoplasmic activation of P21, but not cell senescence, participate in a redundant fashion in embryonic morphogenetic cell death
Source: Cell Death Dis. 2023 Dec 9;14(12):813. doi: 10.1038/s41419-023-06326-6 (PMC10710412; doi:10.1038/s41419-023-06326-6)
Supplement: Supplementary file 1 — Supplementary Table 1 [file 41419_2023_6326_MOESM1_ESM.docx]

| **Supplementary Table 1** | |
| --- | --- |
| **Gene** | **qPCR Primers** |
| *Ch. Cathepsin D* | Fwd: cactgtcacctgctagacatcg |
|  | Rev: cagtgccattctccacatagg |
| *Ch. GLB1* | Fwd: tgtcacagcagcattcttagc |
|  | Rev: acaacaatgtgacggtgtcc |
| *Ch. MMP2* | Fwd: atgatgatgaccgcaagtgg |
|  | Rev: tcctcggagtgctctaatcc |
| *Ch. MMP9* | Fwd: caatgatggcaagctgtgg |
|  | Rev: tcctcggagtgctctaatcc |
| *Ch. IGF1* | Fwd: ccagcagtagacgcttacacc |
|  | Rev: ctcctcaggtcacaactctgg |
| *Ch. IGFBP5* | Fwd: ccttcgttcagtgcgaacc |
|  | Rev: ggtagctcttctcgctgagg |
| *Ch. IGFBP7* | Fwd: cctgagctgtgaagtcattgg |
|  | Rev: gcagaagctccattctctgg |
| *Ch. HGF* | Fwd: gtcaggcaaggagtgtcagc |
|  | Rev: gtctcagcttgccatcagg |
| *Ch. Il6* | Fwd: gcaagaagttcaccgtgtgc |
|  | Rev: ggcaggttgaggttgttcc |
| *Ch. Bak1* | Fwd: ctacgtcaccgaattcatgc |
|  | Rev: aacattgtccagatcgagtgc |
| *Ch. Bcl2* | Fwd:ttgtacggcaacagtatgagg |
|  | Rev: ataagcgccaagagtgatgc |
| *Ch. p21* | Fwd: cgtagaccacgagcagatcc |
|  | Rev: cgtctcggtctcgaagttg |
| *Ch. p16* | Fwd: tggagagcaggacagctacc |
|  | Rev: gcggatgaactagccaacg |
| *Ch. p53* | Fwd: tctggtgagcctcaagatcc |
|  | Rev: caccgtggtacagtcagagc |
| *Ch. p73* | Fwd: cagccacctggacatactcc |
|  | Rev: ggacaccttgatctggatgg |
| *Ch. p63* | Fwd:ggacaccttgatctggatgg |
|  | Rev:tctggtgagcctcaagatcc |
| *Ch. ATG5* | Fwd: ttcgaccaattgcttctgg |
|  | Rev: cgtcttctctccatcttcagg |
| *Ch. ATG7* | Fwd: tgccagtttcttgctgtttg |
|  | Rev: gcaagaggaagctggtcatc |
| *Ch. Sox9* | Fwd: gaggaagtcggtgaagaacg |
|  | Rev: gatgctggaggatgactgc |
| *Ch. GAPDH* | Fwd: ggtggccatcaatgatcc |
|  | Rev: gttctcagccttgacagtgc |

**Supplementary Table 1:** qPCR chicken specific primers.
